# Commentary: “Misguided Effort with Elusive Implications” and “A Multi-Lab Pre-Registered Replication of the Ego Depletion Effect”

**DOI:** 10.3389/fpsyg.2017.00273

**Published:** 2017-02-28

**Authors:** Aaron Drummond, Michael C. Philipp

**Affiliations:** School of Psychology, Massey UniversityPalmerston North, New Zealand

**Keywords:** ego depletion, self control, registered replication report, reanalysis, social psychology

A recent Registered Replication Report (RRR; Hagger et al., 2016) was conducted to examine the ego depletion effect first reported by Baumeister et al. (1998) by replicating Sripada et al.'s (2014) study in 23 international laboratories. Participants completed either an easy or difficult version of a letter “e” task (i.e., Depletion Condition) followed by a multi-source interference task (MSIT) in which participants' reaction time (RT) and reaction time variability (RTV) were recorded as they made congruent and incongruent responses (see https://osf.io/v79xp/ for complete protocol). Ego depletion theory predicts that a sufficiently depleting letter “e” task should result in poorer self-regulation on incongruent MSIT trials (i.e., greater RTs and RTVs). Hagger et al. (2016) found that the ego depletion effect was trivial in effect size and not significantly different from zero. This has called some to question the verisimilitude of ego depletion theory (e.g., Engber, 2016). Yet, several commentaries have also argued that Hagger et al's procedures may be an invalid test of ego depletion (e.g., Baumeister and Vohs, 2016; Sripada et al., 2016; c.f., Hagger and Chatzisarantis, 2016 for an eloquent reply).

One intriguing criticism from Baumeister and Vohs (2016) is that the depleting letter “e” task used in the RRR procedures did not require the exertion necessary to evoke ego-depletion effects on the MSIT. Although the RRR's depleting letter “e” task was rated as a more effortful, difficult, and frustrating than the easy version (Hagger et al., 2016), the task may not have sufficiently taxed self-regulation. One method to examine if this criticism is plausible is to test the underlying process of ego-depletion theory using the existing RRR data.

A recent reanalysis of the Hagger et al.'s (2016) data attempted to address some of Baumeister and Vohs's (2016) concerns by employing a moderation analysis to investigate whether ego depletion was evident in participants who reported exerting more effort on the depleting letter “e” task (Dang, 2016). Although intriguing, Dang's (2016) reanalysis is problematic on three fronts. First, Baumeister et al.'s (1998) ego depletion model theorizes a process by which more challenging, habit-breaking tasks *cause* depleted self-regulation, and that depletion, in turn, *causes* ego depletion effects. In other words, effort should be a *mediating* variable. A moderation model is therefore problematic: It doesn't consider depleted self-regulation as the *process* through which ego depletion occurs, instead specifying self-regulation (indexed via *effort*) as an additional independent factor. Second, Baumeister and Vohs (2016) suggest that *fatigue* is the closest index of depleted self-regulation used in the RRR^1^—not *effort* as examined by Dang (2016). Finally, the reanalysis used an *Effort* × *Depletion Condition* interaction term to predict changes in dependent measures. However, participants' effort ratings significantly differed by depletion condition in the RRR. There is significant collinearity between effort and depletion condition, violating the little-to-no collinearity assumption for regression analyses (e.g., Tabachnick et al., 2007). In addition to being logically problematic, statistically the collinearity between the IV and the moderator results in less stable estimates of the moderator's effect. In light of these issues, we undertook a process-focussed reanalysis of Hagger et al.'s (2016) data to investigate whether the RRR's seeming lack of ego depletion effects may be attributed to an insufficiently depleting letter “e” task, with a particular interest in the indirect effect of the manipulation through self-reported fatigue.

We conducted a series of mediation analyses on Hagger et al's RRR data using the PROCESS macro (Hayes, 2013; model 4; Figure S1) to test whether there was a significant indirect effect of depletion condition on the dependent measures of RT and RTV *through* participants' self-reported fatigue, effort, difficulty, or frustration on the letter “e” task. A bootstrapped mediation analysis is advantageous because it can detect an indirect effect of depletion condition on the dependent measures through a mediator even in the absence of a direct or total effect. This analysis can detect whether the depletion manipulation produced an insuffiecient change in self-regulation for ego depletion effects to occur.

The raw data for each of the 23 participating laboratories was downloaded from the OSF website (https://osf.io/jymhe/), allowing a complete reanalysis of the dependent measures and self-report measures. For all but one laboratory (Schlinkert et al.), we were able to source self-report ratings of fatigue, effort, difficulty, and frustration on the letter “e” task (2,059 participants in total). We did not detect any ratings beyond the 1–7 range of the scales and used the same exclusion criteria for the analysis as were applied in the original RRR.

We undertook bootstrapped mediation analyses (50,000 bootstraps^2^) to give the greatest chance of detecting an indirect effect of the depletion condition on the dependent measures (RT and RTV) via participants' self reported fatigue, effort, difficulty, and frustration scores. As in the original RRR, there was no significant direct or total effect of depletion condition on RT or RTV, *p*s > 0.46. Table 1 presents results of the mediation analyses. Small, positive, significant indirect effects were observed on RT and RTV for all mediators except effort and task difficulty, which only showed a significant indirect effect for RTV. The fatigue mediator shows the smallest indirect effect, and this is the construct that Baumeister and Vohs (2016) suggest is the best indicator of the theorized self-regulation mediator.

**Table 1:** **Indirect effects of task depletion condition on RT and RTV via self-report measures**.

**Indirect effect through**	**Reaction time**		**Reaction time variability**	
	**ß**	**K^2^**	**ß**	**K^2^**
Fatigue	0.0017 [0.0000, 0.0053]	0.002	0.0021 [0.0002, 0.0061]	0.002
Effort	0.0091 [−0.0067, 0.0252]	0.009	0.0191 [0.0035, 0.0351]	0.018
Difficulty	0.0384 [−0.0004, 0.0774]	0.029	0.0391 [0.009, 0.0776]	0.030
Frustration	0.0209 [0.0040, 0.0382]	0.020	0.0317 [0.0144, 0.0495]	0.030

We find mixed support for the assertion that Hagger et al.'s (2016) task was insufficient to produce the ego depletion effect. Although we find a significant ego depletion effect operating through the mediator of fatigue on both RT and RTV, the effect sizes were trivial (Cohen, 1988). Indirect effects through effort, difficulty, and frustration were larger. However, Baumeister and Vohs (2016) suggest that effort, difficulty, and frustration are not necessarily indicative of changes in self-regulation, so these indirect effects may suggest that the RRR manipulation affected something other than self-regulation. We concur with suggestions (e.g., Baumeister and Vohs, 2016; Hagger et al., 2016) that further replication attempts will be critical for understanding and documenting the boundary conditions and processes that lead to ego depletion effects in laboratory settings.

## Author contributions

Conceived and designed the reanalysis: AD and MP. Analysed the data: AD. Wrote the paper: AD and MP.

### Conflict of interest statement

The authors declare that the research was conducted in the absence of any commercial or financial relationships that could be construed as a potential conflict of interest.

## Footnotes

^1^In his review of this commentary Baumiester clarified that of the self-report measures used in the RRR, “fatigue is the most direct characterization of the depleted state that exerting oneself to change oneself should produce.”

^2^Results were qualitatively similar when fewer (e.g., 5,000) bootstraps were used.

## References

[B1] Baumeister R. F. Bratslavsky E. Muraven M. Tice D. M. (1998). Ego depletion: is the active self a limited resource? J. Pers. Soc. Psychol. 74:1252. 10.1037/0022-3514.74.5.1252 9599441

[B2] Baumeister R. F. Vohs K. D. (2016). Misguided effort with elusive implications. Perspect. Psychol. Sci. 11, 574–575. 10.1177/1745691616652878 27474143

[B3] Cohen J. (1988). Statistical Power Analysis for the Behavioural Sciences, 2nd Edn. Hillsdale, NJ: Lawrence Erlbaum Associates.

[B4] Dang J. (2016). Commentary “A multi-lab pre-registered replication of the ego-depletion effect”. Front. Psychol. 7:1155. 10.3389/fpsyg.2016.01155 27535004 PMC4971805

[B5] Engber D. (2016, 3 6). Everything is crumbling. Slate. Available online at: http://www.slate.com/articles/health_and_science/cover_story/2016/03/ego_depletion_an_influential_theory_in_psychology_may_have_just_been_debunked.html

[B6] Hagger M. S. Chatzisarantis N. L. (2016). Commentary: Misguided effort with elusive implications, and sifting signal from noise with replication science. Front. Psychol. 7:621. 10.3389/fpsyg.2016.00621 27561304 PMC4987495

[B7] Hagger M. S. Chatzisarantis N. L. D. Alberts H. Angonno C. O. Batailler C. Birt A.. (2016). A multi-lab pre-registered replication of the ego-depletion effect. Perspect. Psychol. Sci. 11, 546–573. 10.1177/1745691616652873 27474142

[B8] Hayes A. F. (2013). Introduction to Mediation, Moderation, and Conditional Process Analysis. New York, NY: The Guilford Press.

[B9] Sripada C. Kessler D. Jonides J. (2014). Methylphenidate blocks effort-induced depletion of regulatory control in healthy volunteers. Psychol. Sci. 25, 1227–1234. 10.1177/0956797614526415 24756766 PMC4206661

[B10] Sripada C. Kessler D. Jonides J. (2016). Sifting signal from noise with replication science. Perspect. Psychol. Sci. 11, 576–578. 10.1177/1745691616652875 27474144

[B11] Tabachnick B. G. Fidell L. S. Osterlind S. J. (2007). Using Multivariate Statistics, 5th Edn. Boston, MA: Pearson.

