# Supplementary material for: Commentary: “Misguided Effort with Elusive Implications” and “A Multi-Lab Pre-Registered Replication of the Ego Depletion Effect”
Source: Front Psychol. 2017 Feb 28;8:273. doi: 10.3389/fpsyg.2017.00273 (PMC5329006; doi:10.3389/fpsyg.2017.00273)

Figure S1. The mediational models tested in the present reanalysis. The critical pathway is the indirect effect of Depletion Condition on the dependent measures as mediated by participants' self reports of difficulty (depicted), effort, fatigue, and frustration.

.

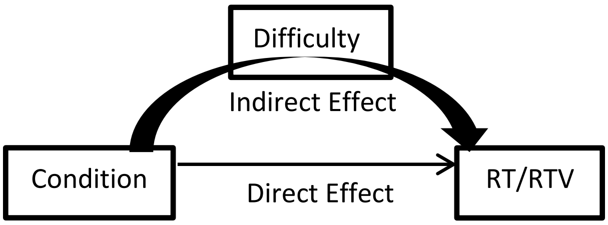

Supplement: Supplementary file 1 [file Image1.PDF]
